# Cognitive profile in burning mouth syndrome versus mild cognitive impairment: A comparative study

**DOI:** 10.1111/odi.15087

**Published:** 2024-07-30

**Authors:** Grazia Daniela Femminella, Federica Canfora, Gennaro Musella, Gianluca Scotto Di Tella, Lorenzo Ugga, Giuseppe Pecoraro, Stefania Leuci, Noemi Coppola, Natascia De Lucia, Nelson Mauro Maldonato, Simone Liguori, Massimo Aria, Luca D'Aniello, Giuseppe Rengo, Michele Davide Mignogna, Daniela Adamo

**Affiliations:** ^1^ Department of Translational Medical Sciences University of Naples “Federico II” Naples Italy; ^2^ Department of Neuroscience, Reproductive Sciences and Dentistry University of Naples “Federico II” Naples Italy; ^3^ Department of Clinic and Experimental Medicine University of Foggia 71122 Foggia Italy; ^4^ Department of Advanced Biomedical Sciences University of Naples “Federico II” Naples Italy; ^5^ Department of Economics and Statistics University of Naples “Federico II” Naples Italy; ^6^ Department of Social Sciences University of Naples “Federico II” Naples Italy

**Keywords:** anxiety, burning mouth syndrome, depression, mild cognitive impairment, MMSE, pain, quality of life

## Abstract

**Objectives:**

This study aims to assess and contrast cognitive and psychological aspects of patients with burning mouth syndrome (BMS‐MCI) and geriatric patients (G‐MCI) with mild cognitive impairment, focusing on potential predictors like pain, mood disorders, blood biomarkers, and age‐related white matter changes (ARWMCs).

**Methods:**

The study enrolled 40 BMS‐MCI and 40 geriatric G‐MCI, matching them by age, gender, and educational background. Participants underwent psychological, sleepiness, and cognitive assessment including the Mini‐Mental State Exam (MMSE), Trail Making Test (TMT), Corsi Block‐Tapping Task, Rey Auditory Verbal Learning Test, Copying Geometric Drawings Test, Frontal Assessment Battery, and Digit Cancellation Test.

**Results:**

G‐MCI patients exhibited higher ARWMCs scores in right (*p* = 0.005**) and left (*p* < 0.001**) temporal regions, which may relate to specific neurodegenerative processes. Conversely, BMS‐MCI patients showed higher levels of depression and anxiety and lower MMSE scores(*p* < 0.001**), also struggling more with tasks requiring processing speed and executive function, as evidenced by their higher TMT‐A scores (*p* < 0.001**).

**Conclusions:**

The study highlights particular deficits in global cognition and processing speed for BMS‐MCI. The influence of educational background, pain levels, cholesterol, sleep disturbances, and anxiety on these cognitive assessments underscores the need for personalized therapeutic strategies addressing both cognitive and emotional aspects of MCI.

## 1 INTRODUCTION

Burning mouth syndrome (BMS) stands as a complex and often enigmatic orofacial disorder. Characterized by a persistent, chronic pain that manifests as a burning sensation in the mouth, BMS is idiopathic in nature, often eluding clear diagnostic markers (“International Classification of Orofacial Pain, 1st edition (ICOP),” 2020). Patients often describe this discomfort as an oral burning sensation, usually bilateral but sometimes also unilateral, following the distributions of one or more branches of the trigeminal, lasting for over 3 months, without any identifiable local or systemic pathological changes (Adamo et al., 2023; Adamo & Spagnuolo, 2022). Accompanying symptoms include xerostomia, taste disturbances, and intraoral foreign body sensation, alongside itching, and tingling. (Adamo et al., 2023; Lamey et al., 2005).

Globally, BMS shows a prevalence of 1.73% among the general population, increasing up to 7.72% in clinical settings of dental patients as outlined in Wu et al.'s meta‐analysis (Wu et al., 2021).

A gender‐based analysis reveals a notable disparity: in the general population, 1.15% of females suffer from BMS, a rate significantly higher than the 0.38% observed in males, indicating a female‐to‐male ratio of approximately 3:1 (Calabria et al., 2024). Age‐wise, BMS predominantly affects individuals aged 50 and above (3.31%), a trend potentially attributable to various factors like hormonal changes, nutritional deficiencies, and cumulative environmental impacts over time (Coculescu et al., 2014). Recent studies point to an age shift in BMS onset, with a higher prevalence around the age of 65, highlighting the evolving landscape of BMS epidemiology in the context of an aging population (Suzuki et al., 2010; Wu et al., 2021).

The etiopathogenesis seems to be multifactorial in which peripheral and central neuropathy and psychological factors play a role in the onset of the disease (Adamo et al., 2020; Shinoda et al., 2019). Dysfunction in the brain networks involving pain matrix areas with different activation patterns of the brain in the anterior cingulate gyrus, bilateral thalamus, left lingual gyrus, bilateral precuneus, right middle frontal gyrus, right pre‐central gyrus, and right inferior semilunar lobule of the cerebellum has been found in BMS patients compared with controls (Kurokawa et al., 2021; Wada et al., 2017). Moreover, BMS patients exhibit a decrease in gray matter volume of the prefrontal cortex, and a higher frequency of white matter changes (WMCs) in specific brain areas suggesting a premature aging of the brain (Adamo et al., 2022; González‐Roldán et al., 2020). These alterations, alongside microglial overactivation and neuroinflammation, may impact cognitive functions in BMS patients (Chen et al., 2024).

Recently, mild cognitive impairment (MCI) has been identified in BMS patients, showing a decrease in global cognitive functions, selective attention, sustained attention, cognitive flexibility, working memory, and executive functions, while verbal memory and praxis constructive skills were preserved in comparison with controls (Canfora et al., 2021).

MCI, the symptomatic pre‐dementia stage, represents a middle ground between normal cognitive aging and dementia‐associated changes. MCI patients present an objective impairment in cognition that is not severe enough to require help with the usual activities of daily living (Fuentes‐Abolafio et al., 2020; Langa & Levine, 2014). Its early detection is crucial, especially in the elderly, to identify populations at higher risk of dementia, particularly Alzheimer's disease (AD) (Batum et al., 2015).

MCI frequently overlaps with mood disorders such as anxiety, depression, and sleep disturbances common also in BMS patients (Goulabchand et al., 2022; Ma, 2020). Indeed, a complex interplay between chronic pain, cognitive decline, and psychological disturbances has been suggested (Chen et al., 2023), because chronic pain further increases the risk for psychiatric vulnerability and social isolation that in turn may be a potential risk factor for cognitive decline in older adults (Bannon et al., 2021).

Sociodemographic variables, systemic diseases, and biochemical markers significantly impact brain health and functionality, making them crucial predictors of cognitive impairments (Popiołek et al., 2020). Factors like age, education level, socioeconomic status, and ethnicity influence the likelihood of cognitive issues due to their association with lifestyle, healthcare access, and environmental exposure (Wang et al., 2023). For instance, older age increases the risk of cognitive decline, while higher education might protect against it. Systemic diseases like cardiovascular conditions and diabetes impair brain function by affecting blood flow and glucose levels, potentially damaging brain cells and leading to cognitive decline (Peters, 2006).

At the molecular level, biochemical markers provide valuable insight into brain processes. Inflammation markers indicate underlying processes that could lead to brain damage, while neurotrophic factors affect neuron survival and health (Lima Giacobbo et al., 2019).

By understanding these predictors, early interventions can be implemented to manage cognitive impairments more effectively, potentially improving outcomes for individuals at risk.

Moreover, patients living with BMS have difficulty adequately diverting attention and memory resources away from pain‐related sensations, feelings, and thoughts, leaving fewer cognitive resources for other concurrent cognitive processes (Adamo et al., 2020; Canfora et al., 2021, 2022; Matsuoka et al., 2010). Consequently, these patients may suffer from specific cognitive impairment in cognitive domains compared with subjects without pain matched for age, gender, and education.

The cognitive impairments observed in BMS patients are notably similar to those identified in patients with MCI, suggesting a potentially significant overlap between these two conditions that warrant further investigation. BMS, typically characterized by chronic pain and discomfort, has been linked to neurological changes such as decreased grey matter volume and alterations in brain networks involved in pain processing. These neurological changes may contribute to the cognitive deficits seen in BMS patients, including issues with attention, memory, and executive functioning.

Comparing these impairments with MCI—a recognized pre‐dementia stage—helps clarify the cognitive decline associated with BMS and may reveal shared neurodegenerative pathways or specific cognitive areas affected by BMS. This comparison underscores the need for cognitive evaluations in BMS patients, enhancing early detection and leading to more effective, comprehensive treatment plans that address both the neurological and psychological aspects of BMS.

Therefore, the primary aim of this study was as follows:

- to compare the cognitive profile of BMS patients with MCI (BMS‐MCI) and geriatric patients with MCI (G‐MCI) but without BMS to evaluate differences in the scores of the tests.

The secondary aims were as follows:

- to evaluate sociodemographic variables, systemic diseases, risk factors, biochemical blood markers, psychological profile, quality of life, and the age‐related white matter changes score (ARWMCs) for two groups
- to analyze potential predictors of cognitive impairment for BMS‐MCI and G‐MCI taking into account sociodemographic variables, systemic diseases, risk factors, biochemical blood markers, psychological profile, and ARWMCs.

## 2 METHODS

A case–control study was carried out over a period from February 2022 to November 2023 at the Oral Medicine and Geriatric Department of the University of Naples “Federico II.” This study followed the ethical principles outlined in the World Medical Association Declaration of Helsinki and received approval from the University's Ethical Committee (Approval Number: 251/19). Moreover, the research methods adhered to the Strengthening the Reporting of Observational Studies in Epidemiology (STROBE) guidelines for observational studies (von Elm et al., 2014).

Patients aged between 55 and 80 years with BMS and geriatric (G) patients, both with MCI were involved as participants in the study during the initial consultation with an oral medicine specialist or a geriatrician.

**FIGURE 1:**
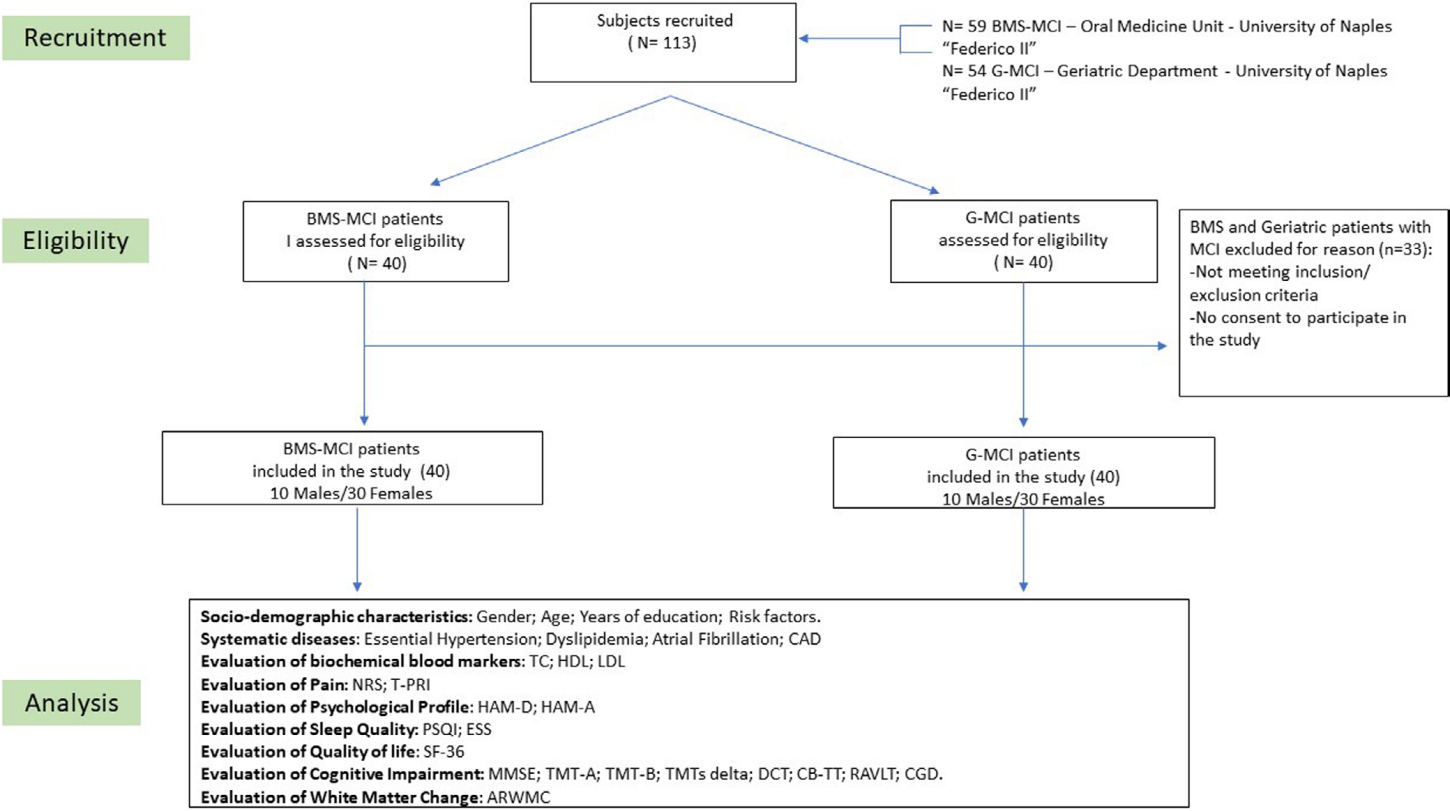
Flowchart of the study. ARWMC, Age‐Related White Matter Change; BMS, Burning mouth syndrome; CAD, Coronary Artery Disease; CB‐TT, Corsi Block‐Tapping Task; CGD, Copying Geometric Drawings; DCT, Digit Cancellation Test; ESS, Epworth Sleepiness Scale; FAB, Frontal Assessment Battery; G, Geriatric; HAM‐A, Hamilton Rating Scale for Anxiety; HAM‐D, Hamilton Rating Scale for Depression; HDL, High‐density lipoprotein; LDL, Low‐density lipoprotein; MCI, mild cognitive impairment; MMSE, Mini‐Mental State Examination; NRS, Numeric Pain Rating Scale; PSQI, Pittsburgh Sleep Quality Index; RAVLT, Rey Auditory Verbal Learning Test; SF‐36, 36 items Short Form Survey; TC, Total cholesterol; TMTs, Trial Making Tests; T‐PRI, Short‐form McGill Pain Questionnaire.

Initially, 59 patients BMS‐MCI and 54 in the G‐MCI patients were enrolled. However, only 40 individuals in each group met the inclusion and exclusion criteria. The study's flowchart, summarizing participant progression, is illustrated in Figure 1. Both groups were matched for age, gender, and educational level. Written informed consent was obtained from all subjects involved in the study and no financial compensation was given for participation.

### 2.1 Inclusion and exclusion criteria

The BMS group's inclusion criteria aligned with the International Classification of Orofacial Pain (“International Classification of Orofacial Pain, 1st edition (ICOP),” 2020) were patients:

- experiencing the symptoms of oral burning recurring daily for >2 h/day for >3 months without any clinical mucosal alterations,
- aged between 55 and 80,
- with normal blood test findings (including blood count, blood glucose levels, and glycated haemoglobin, serum iron, ferritin, folic acid, and transferrin)
- who are currently not in treatment with psychotropic drugs, and patients without any contraindications to MRI scanning (e.g., pacemakers or other metal objects).
- BMS patients with MCI diagnosis according to the NIA‐AA criteria (Albert et al., 2011; De Lucia et al., 2023): patient's concern regarding a cognitive complaint with objective impairment in one or more cognitive domains, preserved independence in activities of daily living, absence of dementia (Winblad et al., 2004).

The inclusion criteria for the G patients were patients:

- with diagnosis of MCI according to the NIA‐AA criteria (Albert et al., 2011; De Lucia et al., 2023): patient's concern regarding a cognitive complaint with objective impairment in one or more cognitive domains, preserved independence in activities of daily living, absence of dementia (Winblad et al., 2004).
- aged between 55 and 80,
- who are currently not in treatment with psychotropic drugs, and patients without any contraindications to MRI scanning (e.g., pacemakers or other metal objects).

The exclusion criteria for both groups were patients:

- suffering from diseases that could be recognized as a causative factor of BMS
- aged <55 and more than 80 years
- unable to understand the questionnaires
- having a history of a psychiatric disorder or an organic brain disorder
- undergoing the treatment with psychotropic drugs
- having a history of alcohol or substance abuse
- in treatment with systemic drugs possibly associated with oral symptoms, and patients suffering from obstructive sleep apnea syndrome (OSAS)

### 2.2 Clinical assessment

Sociodemographic data were systematically examined for each group, including gender, age, and years of education. Additionally, parameters such as risk factors (current smoking status) and the presence of systemic diseases (essential hypertension, dyslipidemia, atrial fibrillation, and coronary artery disease) were evaluated.

For diagnoses of systemic diseases, the patient was considered to suffer from: essential hypertension if she/he had blood pressure (BP) higher than 140/90 mm Hg (SBP/DBP), according to the current ACCF/AHA criteria for uncomplicated hypertension in the elderly or if she/he was taking antihypertensives (Messerli et al., 2011); hypercholesterolemia using the cut‐offs for the metabolic syndrome recommended by the NCEP‐ATPIII (Welty, 2001); atrial fibrillation or coronary artery disease (CAD) if the subject exhibited medical documentation certifying a specific diagnosis after admission.

### 2.3 Neurocognitive and psychological assessment

The neuropsychological assessment was conducted using a battery of scales designed to explore the neurocognitive and psychological profiles and was carried out in a designated hospital room, ensuring standardized clinical procedures.

For neurocognitive evaluation, every participant underwent an in‐depth, one‐to‐one, comprehensive cognitive assessment, following standardized protocols. This assessment aimed to evaluate various cognitive domains, including global mental status, attention, processing speed, working memory, verbal memory, praxis‐constructive skills, and executive functions. The cognitive battery test was administered by the neuropsychologists on the team [GSdT and NDL] and took about 80 min to be administered. It consisted of the:

- Mini‐Mental State Exam (MMSE) (Larner, 2018)
- Trail Making Test (TMT) part A, part B and Delta (Bowie & Harvey, 2006)
- Corsi Block‐Tapping Task (CB‐TT) (Kessels et al., 2000)
- Rey Auditory Verbal Learning Test (RAVLT) (King et al., 1998)
- Copying Geometric Drawings Test (CGD) (Gainotti & Trojano, 2018)
- Frontal Assessment Battery (FAB) (Appollonio et al., 2005)
- Digit Cancellation Test (DCT) (Sala et al., 1992) (Table S3).

Following the results of the neurocognitive assessment, subjects were also classified into amnestic or non‐amnestic MCI, based on prevalent memory impairment, and multiple or single domain if other cognitive domains were also affected (Petersen, 2016).

To identify the psychological profile, this study employed various standardized assessments to estimate the levels of anxiety and depression, health‐related quality of life, and sleep evaluation in both groups. All questionnaires were administered by a psychiatrist from the team [G.P.].

Depressive and anxiety symptoms were assessed using the Hamilton Depression Rating Scale (HAM‐D) and the Hamilton Anxiety Rating Scale (HAM‐A), respectively (Hamilton, 1959, 1967). The 36‐Item Short Form Survey (SF‐36) was performed to assess HRQoL (Lins & Carvalho, 2016, p. 36). The Pittsburgh Sleep Quality Index (PSQI), and the Epworth Sleepiness Scale (ESS), were used to measure the quality of sleep and daytime sleepiness respectively (Buysse et al., 1989; Johns, 1991). In addition, the numeric rating scale (NRS), and the short form of the McGill Pain Questionnaire (SF‐MPQ), were used to evaluate the intensity and quality of pain, respectively (Boonstra et al., 2016; Melzack, 1987) (Table S1).

### 2.4 Laboratory evaluation and magnetic resonance imaging of the brain

The laboratory test was analyzed from a venous blood sample obtained in the morning after a fasting period of about 12 h. The samples were collected in the morning between 8 and 10 a.m. and all biochemical analyses were performed with a Roche Modular Analytics System in the Central Biochemistry Laboratory of our Institution.

In the BMS group, blood glucose levels, glycated hemoglobin, serum iron, ferritin, and transferrin were also evaluated to exclude patients with alterations. The levels of blood lipids [total cholesterol (TC), low‐density lipoprotein cholesterol (LDL), high‐density lipoprotein cholesterol (HDL), and triglycerides (TG)] were evaluated for both groups.

All subjects underwent to Magnetic Resonance Imaging of the brain (MRI) using a 1.5 T Philips Gyroscan Achieva MRI System (Philips, Best, The Netherlands). The methodology comprised two sequences: a turbo spin echo T2‐weighted sequence (TR: 4400 ms, TE: 100 ms, matrix: 256 × 192, slice thickness: 5 mm) and a fluid‐attenuated inversion recovery sequence (TR: 8005 ms, TE: 100 ms, TI: 2200 ms).

Following the criteria from the original study of the ARWMCs, we defined white matter changes (WMCs) as ill‐defined hyperintensities measuring ≥5 mm on both T2‐weighted and FLAIR images (Wahlund et al., 2001). The ARWMC scale categorizes WMCs severity in 10 different brain regions: right and left frontal (RF and LF) lobe, parieto‐occipital region (RPO; LPO), temporal lobe (RT and LT), infratentorial (RIT and LIT) region, and basal ganglia (BG; striatum, globus pallidus, thalamus, internal/external capsule, and insula). The scoring system is described in Table S2.

Using the clinically blinded ARWMC score, two neuroradiologists [R.C., L.U.], evaluated the degree of white matter alterations WMCs in study participants.

### 2.5 Statistical analysis

Statistical analyses were conducted with R software (v. 4.3.1). We used descriptive statistics for sociodemographic and clinical data analysis, applying Pearson Chi‐Square or Fisher's exact test for significant differences in group percentages, with the latter for small sample sizes (cells <5). The Mann–Whitney *U* test compared group median values, including cognitive scores and ARWMC, employing the Bonferroni correction for multiple comparisons. Variable normality was checked via the Shapiro–Wilk test, indicating the necessity for nonparametric tests given non‐normal distributions. Spearman's test assessed correlations between cognitive tests and various predictors like blood markers and ARWMC, with significance considered at *p* < 0.05. Dependencies on qualitative variables (e.g., gender, hypertension) were also examined using the Mann–Whitney *U*‐test. Linear regression analyses, incorporating significant variables from correlations and dependency checks, calculated beta coefficients, standard errors, and adjusted *R* ^2^ values to evaluate the models' fit, covering both BMS‐MCI and G‐MCI groups. Post hoc power analysis for the Mann–Whitney test, considering the Bonferroni adjustment, confirmed the study's power to detect differences, with effect sizes ranging from 0.69 to 0.76 and power levels between 0.91 and 0.97, validating the statistical approaches and findings.

## 3 RESULTS

Figure 1 illustrates the study's flowchart, covering the recruitment, eligibility, and analysis phases. It details the selection process for patients and controls. Initially, 113 subjects were recruited (59 BMS‐MCI and 54 G‐MCI). After assessing eligibility, 40 BMS‐MCI and 40 G‐MCI subjects were included in the study, with 30 females and 10 males in each group.

### 3.1 Sociodemographic and clinical characteristics

Sociodemographic and clinical characteristics (Table 1), including risk factors and biochemical markers, showed no significant differences between BMS‐MCI and G‐MCI patients. Analysis revealed higher ARWMC scores in G‐MCI for both RT (*p* = 0.005**) and LT (*p* < 0.001**) areas. Depression and anxiety levels, measured by HAM‐D and HAM‐A, were significantly higher in BMS‐MCI (*p* < 0.001**), while sleep quality (PSQI) and daytime sleepiness (ESS) showed no group differences. SF‐36 assessments indicated better mental health in G‐MCI (*p* = 0.004*), with no differences in other quality of life measures.

**TABLE 1:** Sociodemographic characteristics, prevalence of systemic diseases, and drug consumption, analysis of the biochemical blood markers and ARWMC scores of BMS‐MCI and G‐MCI patients.

Demographic variables	BMS‐MCI	G‐MCI	*p*‐value
	Frequency (%)	Frequency (%)	
Gender			
Male	10 (25)	10 (25)	1.000
Female	30 (75)	30 (75)	

	Mean ± SD	Mean ± SD	
Age (in years)	67 ± 8.11	67.8 ± 6.46	0.627
Education (in years)	9.33 ± 5.3	11.2 ± 4.85	0.098

	Frequency (%)	Frequency (%)	
Risk factors			
Smoking	10 (25)	8 (20)	0.790
Systemic diseases			
Essential hypertension	19 (47.5)	27 (67.5)	0.113
Dyslipidemia	14 (35)	20 (50)	0.258
Atrial fibrillation	0 (0)	2 (5)	0.494
CAD	2 (5)	6 (15)	0.263

	Median (IQR)	Median (IQR)	
Biochemical blood markers			
TC	174 (153–210)	170 (151–210)	0.977
HDL	55 (45–62.5)	55 (45.8–63.2)	0.725
LDL	126 (121–145)	127 (124–146)	0.647
ARWMC			
RL (Right frontal)	1 (0–1)	1 (1–2)	0.026
LR (Left frontal)	1 (0–1)	1 (0–2)	0.148
RPO (Right parieto‐occipital)	0 (0–1)	0 (0–1)	0.606
LPO (Left parieto‐occipital)	0 (0–1)	0 (0–0)	0.131
RT (Right temporal)	0 (0–0)	1 (0–1)	0.005**
LT (Left temporal)	0 (0–0)	1 (0–1)	<0.001**
RBG (Right basal ganglia)	0 (0–0)	0 (0–0)	0.330
LGB (Left basal ganglia)	0 (0–0)	0 (0–0)	n.a
RI (Right infratentorial)	0 (0–0)	0 (0–0)	n.a
LI (Left infratentorial)	0 (0–0)	0 (0–0)	n.a
Total ARWMC score	2 (0–4.25)	4 (2–6)	0.024

*Note*: IQR is the interquartile range. A significant difference between medians was measured by the Mann–Whitney test.

Abbreviations: ARWMC, Age related white matter changes; BMS‐MCI, Burning mouth syndrome‐Mild cognitive impairment; CAD, Coronary Artery Disease; G‐MCI, Geriatric‐Mild cognitive impairment; HDL, High‐density lipoprotein; LBG, Left basal ganglia; LDL, Low‐density lipoprotein; LF, Left frontal; LI, Left infrantentorial; LPO, Left parieto‐occipital; LT, Left temporal; RBG, Right basal ganglia; RF, Right frontal; RI, Right infratentorial; RPO, Right parieto‐occipital; RT, Right temporal; TC, Total cholesterol.

**Significant with Bonferroni correction 0.005.

A significant difference between the percentages was measured by the Pearson chi‐squared test.

A significance difference between the means was measured by the *t*‐test.

### 3.2 Cognitive performance

Cognitive performance analysis highlighted significant disparities: BMS‐MCI showed a global cognitive decline with lower MMSE scores (Median = 23.35) compared to G‐MCI (Median = 27, *p* < 0.001**), and poorer attention as shown by TMT‐A (Median = 105.5 for BMS‐MCI vs. Median = 74.5 for G‐MCI; *p* < 0.001**). Working memory, assessed by CB‐TT, was similar across groups. Verbal memory, via RAVLT immediate and delayed recall, was significantly lower in BMS‐MCI (Immediate: Median = 38, Delayed: Median = 8) than G‐MCI (Immediate: Median = 25, Delayed: Median = 4.5, *p* < 0.001**). No statistical differences were found in the scores of FAB, CGD, and DCT between the two groups (Table 2).

**TABLE 2:** Level of depression, anxiety, sleep assessment, quality of life, and cognitive evaluation of BMS‐MCI and G‐MCI patients.

	Median (IQR)	Median (IQR)	*p*‐value
Clinical parameter			
HAM‐D	18 (13.75–23.25)	4 (2–9.25)	<0.001**
HAM‐A	17 (15–20.5)	3 (0–7)	<0.001**
PSQI	8.5 (4.75–11)	8 (4–11)	0.794
ESS	5 (3–7.25)	6 (3.75–11)	0.122
SF‐36			
Physical functioning (PF)	60 (43.75–100)	65 (30–90)	0.805
Role physical (RP)	75 (0–100)	25 (0–75)	0.072
Role emotional (RE)	51 (40.75–61)	33.3 (0–100)	0.281
Vitality (VT)	47 (36.5–57)	45 (30–60)	0.405
Mental health (MH)	50 (35–50)	60 (48–72)	0.004*
Social functioning (SF)	62 (46.75–75)	62.5 (37.5–75)	0.601
Bodily pain (BP)	66 (0–100)	55 (25–67.5)	0.967
General health (GH)	48 (40–57)	60 (50–65)	0.028
Cognitive test			
Global cognitive function			
MMSE	23.35 (21.15–25.2)	27 (25–29)	<0.001**
Attention			
TMT‐A	105.5 (80.5–153.25)	74.5 (57.5–87)	<0.001**
Working memory			
CB‐TT	4 (4–4)	4 (4–5)	0.150
Verbal memory			
RAVLT immediate	38 (30–42.25)	25 (19.5–32)	<0.001**
RAVLT delayed	8 (5.75–9)	4.5 (1–7)	<0.001**
Executive functions			
TMT‐B	214.5 (162.25–263.25)	215.5 (145.25–292)	0.784
TMTs Delta	104 (44.75–156.5)	123 (77.75–179)	0.110
FAB	15 (13.75–16)	15 (11.75–17)	0.861
CGD	12.5 (11–13)	13 (11–14)	0.282
DCT	46 (40.75–49)	40.5 (34–47)	0.014

*Note*: IQR is the interquartile range. A significant difference between medians was measured by the Mann–Whitney test.

Abbreviations: CB‐TT, Corsi Block‐Tapping Task; CGD, Copying Geometric Drawings; DCT, Digit Cancellation Test; ESS, Epworth Sleepiness Scale; FAB, Frontal Assessment Battery; HAM‐A, Hamilton Rating Scale for Anxiety; HAM‐D, Hamilton Rating Scale for Depression; MMSE, Mini‐Mental State Examination; PSQI, Pittsburgh Sleep Quality Index; RAVLT, Rey Auditory Verbal Learning Test; SF‐36, 36 items Short Form Survey; TMTs, Trial Making Tests.

**Significant with Bonferroni correction 0.005.

A significant difference between the percentages was measured by the Pearson Chi‐Squared test.

*Significant 0.01 < *p* ≤ 0.05, **Significant *p* ≤ 0.01.

A significance difference between the means was measured by the t‐test.

*Significant 0.01 < *p* ≤ 0.05, **Significant *p* ≤ 0.01.

These results indicate that BMS‐MCI patients show more severe cognitive and emotional symptoms compared to G‐MCI patients. BMS‐MCI patients have notable cognitive decline, higher levels of depression and anxiety, and greater verbal memory impairments.

### 3.3 Correlation analysis between cognitive tests and quantitative predictors

Table 3 details correlations between cognitive tests and quantitative predictors for BMS‐MCI. TMT‐A is inversely correlated with years of education (ρ = −0.445, *p* = 0.004**), suggesting higher education levels are associated with better performance. Positive correlation between TMT‐Delta and TC levels (ρ = 0.539, *p* < 0.001**), indicating higher TC may impair cognitive flexibility. TMT‐B positively correlated with LF‐ARWMC scores (ρ = 0.352, *p* = 0.026*), suggesting WMCs in LF impact cognitive processing speed. Negative correlation between RAVLT immediate recall and NRS score (ρ = −0.409, *p* = 0.009**), indicating pain severity impacts verbal memory. Positive correlations of TMTs Delta with NRS (ρ = 0.367, *p* = 0.020*), and CB‐TT with ESS scores (ρ = 0.312, *p* = 0.050*), highlighting how pain and sleepiness affect cognitive flexibility and working memory, respectively.

**TABLE 3:** Correlation analysis between cognitive tests and quantitative predictors in patients with BMS‐MCI.

Predictors/Cognitive test in BMS‐MCI	MMSE	RAVLT immediate	RAVLT delayed	CGD	CB‐TT	DCT	TMT‐A	TMT‐B	TMTs Delta	FAB
	ρ (*p*‐value)	ρ (*p*‐value)	ρ (*p*‐value)	ρ (*p*‐value)	ρ (*p*‐value)	ρ (*p*‐value)	ρ (*p*‐value)	ρ (*p*‐value)	ρ (*p*‐value)	ρ (*p*‐value)
Demographic variables										
Age	−0.174 (0.283)	−0.023 (0.888)	−0.064 (0.696)	−0.293 (0.067)	−0.114 (0.484)	−0.151 (0.353)	0.275 (0.086)	0.16 (0.323)	0.04 (0.806)	−0.106 (0.514)
Years of education	0.224 (0.164)	0.093 (0.57)	0.116 (0.477)	0.095 (0.559)	0.026 (0.872)	0.001 (0.996)	−0.445 (0.004**)	−0.277 (0.084)	−0.025 (0.879)	0.358 (0.023*)
Biochemical blood markers										
TC	0.131 (0.419)	0.126 (0.438)	0.224 (0.164)	−0.016 (0.924)	0.022 (0.893)	−0.251 (0.118)	−0.014 (0.933)	0.262 (0.102)	0.539 (<0.001**)	−0.12 (0.46)
HDL	−0.269 (0.094)	−0.103 (0.526)	−0.094 (0.566)	−0.099 (0.545)	−0.062 (0.702)	0.08 (0.624)	−0.076 (0.642)	−0.168 (0.3)	−0.206 (0.203)	0.05 (0.76)
LDL	0.048 (0.767)	0.152 (0.35)	0.128 (0.432)	0.12 (0.461)	0.134 (0.408)	0.046 (0.78)	−0.148 (0.364)	−0.055 (0.737)	0.035 (0.83)	0.362 (0.022)
ARWMC										
RL (Right rontal)	−0.203 (0.209)	−0.23 (0.154)	−0.05 (0.758)	−0.304 (0.057)	−0.33 (0.038)	−0.212 (0.189)	0.189 (0.242)	0.285 (0.074)	0.228 (0.156)	−0.192 (0.236)
LF (Left frontal)	−0.185 (0.253)	−0.216 (0.181)	−0.086 (0.596)	−0.228 (0.157)	−0.257 (0.109)	−0.191 (0.237)	0.245 (0.128)	0.352 (0.026*)	0.23 (0.154)	−0.245 (0.127)
RPO (Right parieto‐occipital)	0.078 (0.633)	−0.228 (0.157)	−0.057 (0.725)	−0.272 (0.089)	−0.017 (0.915)	−0.143 (0.377)	0.202 (0.212)	0.237 (0.141)	0.119 (0.464)	−0.088 (0.59)
LPO (Left parieto‐occipital)	0.076 (0.64)	−0.205 (0.204)	−0.042 (0.796)	−0.258 (0.109)	−0.013 (0.936)	−0.131 (0.419)	0.212 (0.188)	0.248 (0.122)	0.135 (0.405)	−0.092 (0.572)
RT (Right temporal)	0.175 (0.279)	−0.197 (0.224)	−0.075 (0.645)	−0.301 (0.059)	0.024 (0.886)	−0.173 (0.286)	0.078 (0.634)	0.085 (0.602)	0.005 (0.976)	−0.031 (0.849)
LT (Left temporal)	−0.059 (0.716)	−0.068 (0.677)	−0.144 (0.376)	−0.394 (0.012)	0.024 (0.886)	0.017 (0.919)	0.052 (0.748)	−0.021 (0.898)	−0.202 (0.212)	−0.021 (0.898)
RBG (Right basal‐ganglia)	−0.143 (0.378)	−0.236 (0.143)	−0.079 (0.626)	−0.299 (0.061)	−0.198 (0.22)	−0.179 (0.27)	0.229 (0.155)	0.305 (0.055)	0.165 (0.309)	−0.191 (0.237)
Total ARWMC score	0.039 (0.81)	−0.136 (0.401)	−0.078 (0.632)	−0.038 (0.818)	−0.089 (0.586)	0.008 (0.959)	−0.015 (0.929)	−0.025 (0.879)	−0.12 (0.462)	−0.013 (0.936)
NRS	−0.277 (0.084)	−0.409 (0.009**)	−0.211 (0.192)	0.024 (0.882)	−0.292 (0.067)	−0.046 (0.776)	0.013 (0.937)	0.23 (0.154)	0.367 (0.020*)	−0.27 (0.092)
T‐PRI	0.188 (0.246)	−0.071 (0.664)	0.105 (0.520)	0.143 (0.378)	0.253 (0.116)	−0.084 (0.606)	0.106 (0.516)	0.093 (0.569)	−0.011 (0.947)	0.288 (0.071)
HAM‐A	−0.043 (0.795)	−0.221 (0.17)	−0.147 (0.366)	0.252 (0.116)	−0.013 (0.935)	−0.068 (0.677)	0.199 (0.219)	0.129 (0.428)	0.11 (0.5)	−0.07 (0.666)
HAM‐D	−0.041 (0.803)	−0.243 (0.132)	−0.128 (0.431)	0.224 (0.165)	−0.168 (0.301)	−0.043 (0.791)	0.07 (0.669)	0.044 (0.79)	0.058 (0.722)	−0.133 (0.414)
PSQI	0.138 (0.394)	−0.263 (0.101)	−0.229 (0.155)	0.098 (0.549)	0.075 (0.644)	−0.305 (0.056)	0.183 (0.258)	0.188 (0.245)	0.167 (0.304)	0.137 (0.398)
ESS	−0.001 (0.994)	−0.106 (0.514)	0.008 (0.959)	0.312 (0.050*)	0.047 (0.772)	0.191 (0.238)	−0.232 (0.15)	−0.215 (0.183)	−0.036 (0.827)	0.128 (0.431)
SF‐36										
SF‐36 PF	0.022 (0.891)	0.047 (0.773)	0.179 (0.268)	−0.019 (0.909)	0.079 (0.63)	0.193 (0.233)	−0.058 (0.72)	−0.093 (0.568)	−0.001 (0.996)	0.02 (0.901)
SF‐36 PH	−0.192 (0.235)	0.114 (0.483)	0.16 (0.324)	−0.113 (0.489)	0.095 (0.559)	−0.028 (0.865)	−0.015 (0.925)	0.1 (0.541)	0.14 (0.387)	−0.29 (0.07)
SF‐36 RE	0.076 (0.643)	0.123 (0.448)	0.081 (0.62)	0.056 (0.731)	0.03 (0.854)	0.167 (0.304)	−0.25 (0.12)	−0.237 (0.142)	−0.064 (0.694)	0.048 (0.769)
SF‐36 VT	−0.139 (0.392)	0.13 (0.422)	−0.036 (0.825)	0.03 (0.854)	0.057 (0.727)	0.15 (0.357)	−0.126 (0.438)	−0.073 (0.654)	0.029 (0.858)	−0.238 (0.139)
SF‐36 MH	−0.203 (0.21)	0.051 (0.756)	0.025 (0.879)	−0.23 (0.154)	0.055 (0.738)	0.035 (0.829)	−0.03 (0.854)	0.014 (0.934)	0.08 (0.625)	−0.245 (0.127)
SF‐36 SF	0.057 (0.726)	−0.016 (0.924)	−0.134 (0.411)	0.079 (0.628)	−0.001 (0.993)	0.075 (0.646)	−0.243 (0.131)	−0.231 (0.151)	−0.04 (0.808)	−0.004 (0.98)
SF‐36 BP	0.04 (0.807)	0.182 (0.26)	0.116 (0.475)	−0.052 (0.75)	0.213 (0.187)	0.067 (0.68)	−0.118 (0.468)	−0.044 (0.786)	0.052 (0.749)	−0.146 (0.369)
SF‐36 GH	−0.08 (0.622)	−0.038 (0.817)	−0.131 (0.421)	−0.175 (0.279)	0.017 (0.919)	0.098 (0.547)	−0.012 (0.939)	−0.025 (0.88)	0.017 (0.916)	−0.225 (0.163)

*Note*: ρ is Spearman's correlation coefficient. *p*‐value—*Significant 0.01 < *p*‐value ≤0.05. **Significant *p*‐value ≤0.01.

Abbreviations: ARWMC, Age related white matter changes; BMS‐MCI, Burning mouth syndrome‐Mild cognitive impairment; CB‐TT, Corsi Block‐Tapping Task; CGD, Copying Geometric Drawings; DCT, Digit Cancellation Test; ESS, Epworth Sleepiness Scale; FAB, Frontal Assessment Battery; G‐MCI, Geriatric‐Mild cognitive impairment; HAM‐A, Hamilton Rating Scale for Anxiety; HAM‐D, Hamilton Rating Scale for Depression; HDL, High‐density lipoprotein; LBG, Left basal ganglia; LDL, Low‐density lipoprotein; LF, Left frontal; LINF, Left infrantentorial; LPO, Left parieto‐occipital; LT, Left temporal; MMSE, Mini Mental State Examination; PSQI, Pittsburgh Sleep Quality Index; RAVLT, Rey Auditory Verbal Learning Test; RBG, Right basal ganglia; RF, Right frontal; RINF, Right infratentorial; RPO, Right parieto‐occipital; RT, Right temporal; SF‐36 BP, Bodily Pain; SF‐36 GH, General Health; SF‐36 MH, Mental Health; SF‐36 PF, Physical functioning; SF‐36 RE, Role emotional; SF‐36 RP, Role physical; SF‐36 SF, Social Functioning; SF‐36 VT, Vitality; SF‐36, 36 items Short Form Survey; TC, Total cholesterol; TMTs, Trial Making Tests.

These findings suggest that in BMS‐MCI patients, higher education levels correlate with better cognitive test performance, while elevated cholesterol and LF‐WMCs are associated with decreased cognitive flexibility and processing speed. Additionally, pain severity and sleepiness appear to negatively impact verbal memory, cognitive flexibility, and working memory.

Table 4 shows correlations between cognitive tests and quantitative predictors for G‐MCI. A strong positive correlation between CGD scores and years of education (ρ = 0.626, *p* < 0.001**) underscores education's protective role against cognitive decline in spatial abilities. TC levels showed mixed effects: positively correlating with DCT scores (ρ = 0.39, *p* < 0.013*) but negatively with TMT‐A (ρ = −0.618, *p* < 0.001**) and TMT‐B (ρ = 0.315, *p* < 0.048*), suggesting nuanced impacts of cholesterol on cognitive tasks. A negative correlation between ARWMC scores in the RPO region with CGD (ρ = −0.333, *p* < 0.039*) suggesting that a greater degree of WMCs is associated with a worse performance in visual perception and visuomotor coordination, functions that can be affected by brain's health of the parieto‐occipital regions. Similarly, a significant negative correlation between LPO and FAB (ρ: 0.312, *p*‐value <0.05*) has been found suggesting that an increase in the score of WMCs is associated with a decrease in performance on tests that assess executive functions.

**TABLE 4:** Correlation analysis between cognitive tests and quantitative predictors in patients with G‐MCI.

Predictors/cognitive test in G‐MCI	MMSE	RAVLT immediate	RAVLT delayed	CGD	CB‐TT	DCT	TMT‐A	TMT‐B	TMTs Delta	FAB
	ρ (*p*‐value)	ρ (*p*‐value)	ρ (*p*‐value)	ρ (*p*‐value)	ρ (*p*‐value)	ρ (*p*‐value)	ρ (*p*‐value)	ρ (*p*‐value)	ρ (*p*‐value)	ρ (*p*‐value)
Demographic variables										
Age	0.159 (0.326)	−0.289 (0.07)	−0.3 (0.06)	0.258 (0.113)	−0.083 (0.612)	−0.1 (0.538)	0.271 (0.09)	0.045 (0.781)	−0.132 (0.418)	−0.059 (0.717)
Years of education	0.253 (0.116)	0.053 (0.746)	0.016 (0.923)	0.626 (<0.001**)	−0.093 (0.567)	0.198 (0.22)	0.013 (0.938)	−0.18 (0.267)	−0.175 (0.281)	0.154 (0.344)
Biochemical blood markers										
TC	−0.175 (0.28)	0.22 (0.172)	0.166 (0.306)	0.227 (0.165)	0.029 (0.86)	0.39 (0.013*)	−0.618 (<0.001**)	−0.315 (0.048*)	0.045 (0.783)	0.113 (0.488)
HDL	−0.054 (0.742)	0.186 (0.25)	0.058 (0.72)	−0.141 (0.392)	−0.145 (0.371)	−0.097 (0.55)	0.075 (0.643)	−0.011 (0.949)	−0.022 (0.89)	0.072 (0.659)
LDL	0.075 (0.645)	−0.008 (0.962)	0.05 (0.76)	−0.034 (0.836)	0.115 (0.479)	−0.091 (0.576)	0.081 (0.618)	0 (0.998)	0.041 (0.801)	0.152 (0.349)
ARWMC										
RL (Right frontal)	0.085 (0.6)	−0.117 (0.474)	−0.107 (0.511)	0.063 (0.701)	0.061 (0.708)	0.069 (0.674)	−0.021 (0.897)	−0.147 (0.367)	−0.22 (0.172)	0.057 (0.728)
LF (Left frontal)	0.086 (0.597)	−0.121 (0.455)	−0.025 (0.878)	0.057 (0.728)	0.018 (0.913)	0.029 (0.858)	−0.085 (0.602)	−0.14 (0.39)	−0.198 (0.221)	0.088 (0.59)
RPO (Right parieto‐occipital)	−0.292 (0.068)	−0.162 (0.318)	−0.285 (0.075)	−0.333 (0.039*)	−0.155 (0.339)	−0.181 (0.264)	0.046 (0.78)	0.234 (0.147)	0.194 (0.23)	−0.245 (0.128)
LPO (Left parieto‐occipital)	−0.135 (0.406)	−0.138 (0.397)	−0.114 (0.482)	−0.127 (0.442)	−0.265 (0.099)	−0.241 (0.134)	0.198 (0.222)	0.272 (0.09)	0.144 (0.374)	−0.312 (0.05*)
RT (Right temporal)	0.101 (0.534)	−0.021 (0.899)	0.012 (0.943)	0.024 (0.883)	0.058 (0.724)	0.111 (0.497)	−0.039 (0.809)	−0.091 (0.576)	−0.254 (0.113)	−0.095 (0.562)
LT (Left temporal)	0.001 (0.997)	−0.13 (0.422)	−0.082 (0.613)	−0.057 (0.732)	−0.17 (0.294)	−0.046 (0.777)	0.129 (0.427)	0.11 (0.499)	−0.086 (0.597)	−0.122 (0.453)
RBG (Right basal‐ganglia)	−0.098 (0.546)	−0.188 (0.246)	−0.175 (0.28)	0.015 (0.928)	−0.214 (0.185)	−0.215 (0.182)	0.229 (0.155)	0.216 (0.18)	0.104 (0.523)	0.091 (0.577)
Total ARWMC score	0.039 (0.81)	−0.136 (0.401)	−0.078 (0.632)	−0.038 (0.818)	−0.089 (0.586)	0.008 (0.959)	−0.015 (0.929)	−0.025 (0.879)	−0.12 (0.462)	−0.013 (0.936)
HAM‐A	−0.472 (0.002**)	−0.042 (0.798)	−0.038 (0.816)	−0.188 (0.251)	−0.104 (0.523)	−0.167 (0.304)	0.241 (0.134)	0.444 (0.004**)	0.421 (0.007**)	−0.238 (0.139)
HAM‐D	−0.285 (0.075)	0.107 (0.513)	0.052 (0.75)	−0.137 (0.407)	−0.086 (0.598)	0.041 (0.802)	0.005 (0.974)	0.175 (0.281)	0.259 (0.107)	0.029 (0.858)
PSQI	0.047 (0.771)	0.112 (0.493)	0.196 (0.225)	−0.099 (0.548)	−0.392 (0.012*)	0.031 (0.849)	−0.161 (0.322)	−0.08 (0.625)	0.021 (0.899)	0.249 (0.122)
ESS	0.161 (0.322)	0.173 (0.285)	0.156 (0.335)	−0.233 (0.154)	−0.162 (0.318)	−0.021 (0.899)	−0.03 (0.856)	−0.244 (0.13)	−0.318 (0.045*)	0.214 (0.185)
SF‐36										
SF‐36 PF	0.226 (0.178)	−0.201 (0.234)	−0.138 (0.415)	0.364 (0.029*)	0.135 (0.425)	−0.121 (0.477)	0.09 (0.596)	−0.058 (0.733)	−0.07 (0.682)	0.178 (0.292)
SF‐36 PH	−0.002 (0.992)	0.055 (0.745)	−0.064 (0.706)	0.238 (0.162)	0.27 (0.106)	−0.011 (0.949)	−0.075 (0.661)	−0.076 (0.655)	−0.015 (0.929)	0.127 (0.452)
SF‐36 RE	0.202 (0.23)	−0.13 (0.444)	−0.077 (0.652)	0.171 (0.319)	0.33 (0.046*)	−0.008 (0.964)	−0.076 (0.655)	−0.134 (0.428)	−0.095 (0.576)	0.25 (0.136)
SF‐36 VT	0.134 (0.428)	−0.164 (0.333)	−0.179 (0.289)	0.166 (0.333)	0.003 (0.988)	−0.017 (0.92)	0.054 (0.751)	−0.101 (0.551)	−0.236 (0.16)	0.086 (0.613)
SF‐36 MH	0.352 (0.033*)	0.064 (0.706)	−0.013 (0.941)	0.129 (0.452)	0.29 (0.081)	0.102 (0.549)	0.047 (0.784)	−0.274 (0.101)	−0.427 (0.008**)	0.14 (0.41)
SF‐36 SF	0.382 (0.020*)	0.15 (0.377)	0.133 (0.433)	0.033 (0.846)	0.333 (0.044*)	0.168 (0.319)	−0.103 (0.543)	−0.239 (0.154)	−0.119 (0.484)	0.203 (0.228)
SF‐36 BP	0.033 (0.847)	−0.15 (0.376)	−0.196 (0.245)	0.501 (0.002**)	0.198 (0.24)	−0.049 (0.772)	0.062 (0.715)	−0.003 (0.985)	−0.003 (0.984)	0.099 (0.56)
SF‐36 GH	0.021 (0.902)	−0.145 (0.393)	−0.148 (0.383)	0.186 (0.276)	0.201 (0.233)	−0.057 (0.739)	0.071 (0.674)	0.104 (0.539)	0.058 (0.734)	0.128 (0.451)

Abbreviations: ARWMC, Age related white matter changes; CB‐TT, Corsi Block‐Tapping Task; CGD, Copying Geometric Drawings; DCT, Digit Cancellation Test; ESS, Epworth Sleepiness Scale; FAB, Frontal Assessment Battery; G‐MCI, Geriatric‐Mild cognitive impairment; HAM‐A, Hamilton Rating Scale for Anxiety; HAM‐D, Hamilton Rating Scale for Depression; HDL, High‐density lipoprotein; LBG, Left basal ganglia; LDL, Low‐density lipoprotein; LF, Left frontal; LINF, Left infrantentorial; LPO, Left parieto‐occipital; LT, Left temporal; MMSE, Mini Mental State Examination; PSQI, Pittsburgh Sleep Quality Index; RAVLT, Rey Auditory Verbal Learning Test; RBG, Right basal ganglia; RF, Right frontal; RINF, Right infratentorial; RPO, Right parieto‐occipital; RT, Right temporal; SF‐36 BP, Bodily Pain; SF‐36 GH, General Health; SF‐36 MH, Mental Health; SF‐36 PF, Physical functioning; SF‐36 RE, Role emotional; SF‐36 RP, Role physical; SF‐36 SF, Social Functioning; SF‐36 VT, Vitality; SF‐36, 36 items Short Form Survey; TC, Total cholesterol; TMTs, Trial Making Tests.

*Significant 0.01 < *p* ≤ 0.05, **Significant *p* ≤ 0.01.

Additionally, HAM‐A negatively correlated with MMSE scores (ρ = −0.472, *p* < 0.002**) but positively with TMT‐B and TMTs Delta (ρ = 0.444, *p* < 0.004**; ρ = 0.421, *p* = 0.007**), showing anxiety's detrimental effect on global cognition and executive functions. PSQI and ESS showed moderate negative correlations with CB‐TT (ρ = −0.392, *p* < 0.012*) and TMT‐Delta (ρ = −0.318, *p* < 0.045*), respectively, highlighting their impact on cognitive functions.

Among the SF‐36 subitems, moderate positive statistically significant correlation has been found between PF and RAVLT scores (ρ: 0.364; *p*‐value: 0.029*), RE and CB‐TT (ρ: 0.33; *p*‐value: 0.046*), MH and MMSE (ρ: 0.352; *p*‐value: 0.033*), SF and MMSE (ρ: 0.382; *p*‐value: 0.020*), SF and CB‐TT (ρ: 0.333; *p*‐value: 0.044**); BP and CGD (ρ: 0.501; *p*‐value: 0.002**); while a negative correlation between MH and TMTs Delta (ρ: −0.427; *p*‐value: 0.008**) suggesting that individuals with better physical functioning tend to have better short‐term and long‐term verbal memory abilities and who better manage their roles despite emotional problems tend to perform better on visuo‐spatial working memory tasks. Additionally, better mental health status and individuals with better social functioning, or the ability to engage in normal social activities tend to have a better overall cognitive function, less impairment in cognitive flexibility and executive function, and better visuo‐spatial working memory.

These findings indicate that for G‐MCI patients, higher education levels are linked to better spatial cognitive abilities, illustrating its protective effect against cognitive decline. The results also suggest that cholesterol levels can both improve and impair different cognitive tasks. Additionally, anxiety adversely affects both global cognition and executive functions, while poor sleep quality and sleepiness negatively impact specific cognitive abilities.

### 3.4 Dependence analysis between cognitive tests and qualitative predictors

Table 5 analyzes cognitive test scores against qualitative predictors for BMS‐MCI.

**TABLE 5:** Dependence analysis between cognitive tests, and qualitative predictors in patients with BMS‐MCI.

BMS MCI	MMSE		RAVLT immediate		RAVLT delayed		CGD		CB‐TT		DCT		TMT‐A		TMT‐B		TMTs Delta		FAB	
	Median (IQR)	*p*‐value	Median (IQR)	*p*‐value	Median (IQR)	*p*‐value	Median (IQR)	*p*‐value	Median (IQR)	*p*‐value	Median (IQR)	*p*‐value	Median (IQR)	*p*‐value	Median (IQR)	*p*‐value	Median (IQR)	*p*‐value	Median (IQR)	*p*‐value
Gender																				
Female	23.25 [21.2;25.15]	0.743	39.5 [30.75;43]	0.260	8 [5.25;9]	0.764	12 [11;13]	0.162	4 [4;4]	0.262	47 [41.25;49]	0.913	98.5 [81.75;155.25]	0.975	208 [158.5;260.5]	0.274	102 [44.25;154.5]	0.502	15 [14;16]	0.125
Male	24.05 [21.35;25.15]		31.5 [29.25;38.5]		6 [6;8.75]		13 [12;14]		4 [3.62;4.94]		44 [41;49]		111.5 [83.5;127]		251.5 [207;268]		127 [89.25;154.75]		14 [13.25;14.75]	
Essential hypertension																				
Yes	22.7 [20.45;24.8]	0.046*	38 [29.5;42.5]	0.684	8 [5.5;9]	0.575	13 [11;13]	0.655	4 [4;4]	0.678	47 [42.5;48.5]	0.849	153 [91;168]	0.010*	216 [173;346]	0.159	86 [45.5;172]	0.946	14 [13.5;16]	0.592
No	24.3 [22.4;25.2]		39 [30;42]		8 [6;9]		12 [12;13]		4 [4;4]		44 [40;51]		90 [76;114]		204 [149;251]		108 [45;151]		15 [14;16]	
Smoking status																				
Smoker	23.25 [21.4;24.9]	0.988	41.5 [34.75;43.75]	0.080	9 [7.25;9]	0.263	12 [12;13]	0.809	4 [4;4]	0.485	47.5 [40.25;51]	1.000	115 [92;124.5]	0.876	239 [157.75;279]	0.791	130 [42;168.25]	0.553	14.5 [13.25;15.75]	0.669
Not Smoker	23.65 [21.05;25.15]		37 [28.25;41.5]		8 [5;9]		13 [11;13]		4 [4;4]		45 [41.25;49]		98 [79.5;153.75]		213 [167.75;253]		104 [45.5;145.5]		15 [14;16]	
Dyslipidemia																				
Yes	24.8 [20.825;25.15]	0.898	38.5 [33.5;41.5]	0.777	8 [7.25;9]	0.375	12 [11;13]	0.549	4 [4;4]	0.960	45.5 [40.25;48.75]	0.619	103.5 [81.75;145]	0.702	228.5 [167.75;281.25]	0.427	142.5 [74.5;159]	0.275	15 [14;16]	0.527
No	22.95 [21.2;25.15]		37.5 [29.25;42.75]		7.5 [5;9]		13 [11;13]		4 [4;4]		46 [43.25;50.5]		107 [81;147.25]		214.5 [157.5;251.75]		102 [44.25;140.75]		15 [13.25;16]	
Atrial fibrillation																				
Yes	–		–		–		–		–		–		–		–					
No	23.35 [21.15;25.2]	–	38 [30;42.25]	–	8 [5.75;9]	–	12.5 [11;13]	–	4 [4;4]	–	46 [40.75;49]	–	105.5 [80.5;153.25]	–	214.5 [162.25;263.25]	–	104 [44.75;156.5]	–	15 [13.75;16]	–
CAD																				
Yes	23.55 [22.975;24.125]	0.975	41.5 [39.25;43.75]	0.419	8 [8;8]	0.925	11.5 [11.25;11.75]	0.371	4 [4;4]	0.798	39.5 [37.25;41.75]	0.202	140 [133;147]	0.291	256 [242;270]	0.336	100.5 [99.75;101.25]	0.852	15 [14.5;15.5]	0.801
No	23.35 [21.05;25.2]		38 [30;42]		8 [5.25;9]		13 [11;13]		4 [4;4]		47 [41.25;49]		103.5 [79.5;147.25]		213 [160.75;260.5]		106 [44.25;157.5]		15 [13.25;16]	

*Note*: IQR is the interquartile range. Wilcoxon–Mann–Whitney Test. *p*‐value—Significant *p*‐value ≤0.05.

Atrial fibrillation was not measured as a variable in the study because the percentage of patients affected by atrial fibrillation frequency within this group was 0%.

Abbreviations: BMS‐MCI, Burning mouth syndrome‐Mild cognitive impairment; CAD, Coronary Artery Disease; CB‐TT, Corsi Block‐Tapping Task; CGD, Copying Geometric Drawings; DCT, Digit Cancellation Test; FAB, Frontal Assessment Battery; MMSE, Mini‐Mental State Examination; RAVLT, Rey Auditory Verbal Learning Test; TMTs, Trial Making Tests.

Essential hypertension was significantly associated with lower MMSE scores (*p* = 0.046*), suggesting a worse global cognitive function in hypertensive patients. Hypertensive patients also scored higher on TMT‐A (*p* = 0.010*), indicating reduced processing speed or executive function. No significant effects were observed from gender, smoking, dyslipidemia, or CAD on cognitive performances.

In G‐MCI (Table 6) the atrial fibrillation correlated significantly with TMT‐B (*p* = 0.027*) and FAB scores (*p* = 0.036*), implying its potential impact on executive functions and processing speed.

**TABLE 6:** Dependence analysis between cognitive tests, and qualitative predictors in patients with G‐MCI.

G‐MCI	MMSE		RAVLT immediate		RAVLT delayed		CGD		CB‐TT		DCT		TMT‐A		TMT‐B		TMTs Delta		FAB	
	Median (IQR)	*p*‐value	Median (IQR)	*p*‐value	Median (IQR)	*p*‐value	Median (IQR)	*p*‐value	Median (IQR)	*p*‐value	Median (IQR)	*p*‐value	Median (IQR)	*p*‐value	Median (IQR)	*p*‐value	Median (IQR)	*p*‐value	Median (IQR)	*p*‐value
Gender																				
Female	27 [25;28.75]	0.069	22.5 [18.5;32]	0.719	3 [1;7]	0.336	13 [11;14]	0.730	4 [3.25;5]	0.803	41.5 [34;46.75]	0.628	77.5 [54.5;87]	0.662	235 [150.5;298.75]	0.073	151.5 [81.5;197]	0.057	15 [11;16]	0.405
Male	28.5 [27;29]		27.5 [20.5;30.75]		5.5 [3.5;7]		13 [12;13]		4 [4;4.75]		39 [35.75;49.75]		70.5 [59.75;78.25]		165 [120;198.5]		86 [65;116]		15.5 [14.25;17]	
Essential hypertension																				
Yes	27 [25.5;29]	0.827	27 [20.5;30]	0.654	4 [1;6.5]	0.315	13 [11;14]	0.975	4 [3.5;5]	0.927	40 [33.5;45.5]	0.193	79 [64.5;88]	0.129	220 [138;293]	0.908	125 [66.5;194]	0.573	15 [11.5;17]	0.942
No	27 [25;29]		20 [17;35]		6 [3;8]		13 [11.75;14]		4 [4;5]		43 [35;48]		62 [52;76]		211 [150;247]		121 [90;173]		15 [13;16]	
Smoking status																				
Smoker	26 [25;27]	0.161	20.5 [19;24.75]	0.236	2 [0.75;3.75]	0.056	14 [11;14]	0.419	4 [3.75;5]	0.914	34 [27;51.75]	0.388	79.5 [62.75;89]	0.624	280.5 [166.25;296.25]	0.465	151.5 [75.25;204.25]	0.685	14 [10.5;16.25]	0.61
Not smoker	27 [25.75;29]		27.5 [19.5;32.25]		5 [3;7.25]		13 [11.5;13.5]		4 [4;5]		41.5 [35;46.25]		72 [57.5;87]		205 [145.25;255.25]		123 [80;170.75]		15 [12;17]	
Dyslipidemia																				
Yes	27 [25.75;29]	0.902	22 [20;30]	0.735	5 [2.5;7]	0.935	13 [11.75;14]	0.919	4 [4;5]	0.412	40.5 [34;47.25]	0.946	77.5 [61;90.25]	0.387	209.5 [147.5;296.25]	0.796	120.5 [74.5;174]	0.626	14 [12;15.25]	0.226
No	27 [24.75;29]		27 [17.75;32.25]		3 [1;8]		13 [11;14]		4 [3.75;5]		41 [34;47]		70 [55.5;84]		215.5 [143.25;258]		131 [81.75;179]		16 [10.75;17.25]	
Atrial fibrillation																				
Yes	28.5 [27.75;29.25]	0.300	23.5 [20.25;26.75]	0.709	4.5 [3.75;5.25]	1.000	13.5 [13.25;13.75]	0.374	5 [4.5;5.5]	0.276	46 [42;50]	0.384	49 [44;54]	0.145	108.5 [107.25;109.75]	0.027*	59.5 [55.75;63.25]	0.067	18 [18;18]	0.036*
No	27 [25;29]		25 [20;32]		4.5 [1;7]		13 [11;14]		4 [4;5]		40.5 [34;46.75]		76 [59;87]		220 [150;294]		131 [81.25;181]		15 [11.25;16]	
CAD																				
Yes	27 [26.25;27]	0.759	28 [19.75;30.25]	1.000	5.5 [3.5;6.75]	0.774	13.5 [13;14]	0.243	4 [4;4]	0.643	44 [40.25;50.75]	0.178	76 [58.75;86.5]	1.000	200 [157.5;280]	1.000	110.5 [86.75;154.5]	0.970	15.5 [14.25;16.75]	0.675
No	27 [25;29]		22.5 [20;32]		3.5 [1;7]		13 [11;14]		4 [4;5]		39 [34;46]		73.5 [58.25;86.75]		215.5 [141.75;288.25]		131 [75;181]		15 [11.25;16.75]	

*Note*: IQR is the interquartile range. Wilcoxon–Mann–Whitney Test. *p*‐value—*Significant 0.01 < *p*‐value ≤0.05. **Significant *p*‐value ≤0.01.

Atrial fibrillation was not measured as a variable in the study because the percentage of patients affected by atrial fibrillation frequency within this group was 0%.

Abbreviations: CAD, Coronary Artery Disease; CB‐TT, Corsi Block‐Tapping Task; CGD, Copying Geometric Drawings; DCT, Digit Cancellation Test; FAB, Frontal Assessment Battery; G‐MCI, Geriatric‐Mild cognitive impairment; MMSE, Mini‐Mental State Examination; RAVLT, Rey Auditory Verbal Learning Test; TMTs, Trial Making Tests.

Other factors like gender, hypertension, smoking, dyslipidemia, and CAD showed no significant correlation with the range of cognitive tests evaluated.

These results suggest that essential hypertension in BMS‐MCI patients is associated with worse overall cognitive function and slower processing speeds or reduced executive function. Conversely, in G‐MCI patients, atrial fibrillation significantly affects executive functions and processing speed. The findings indicate the importance of managing cardiovascular health to mitigate cognitive decline in patients with MCI, while other factors such as gender, smoking, dyslipidemia, and CAD do not show a significant impact on cognitive performance in these groups.

### 3.5 White matter changes

**FIGURE 2:**
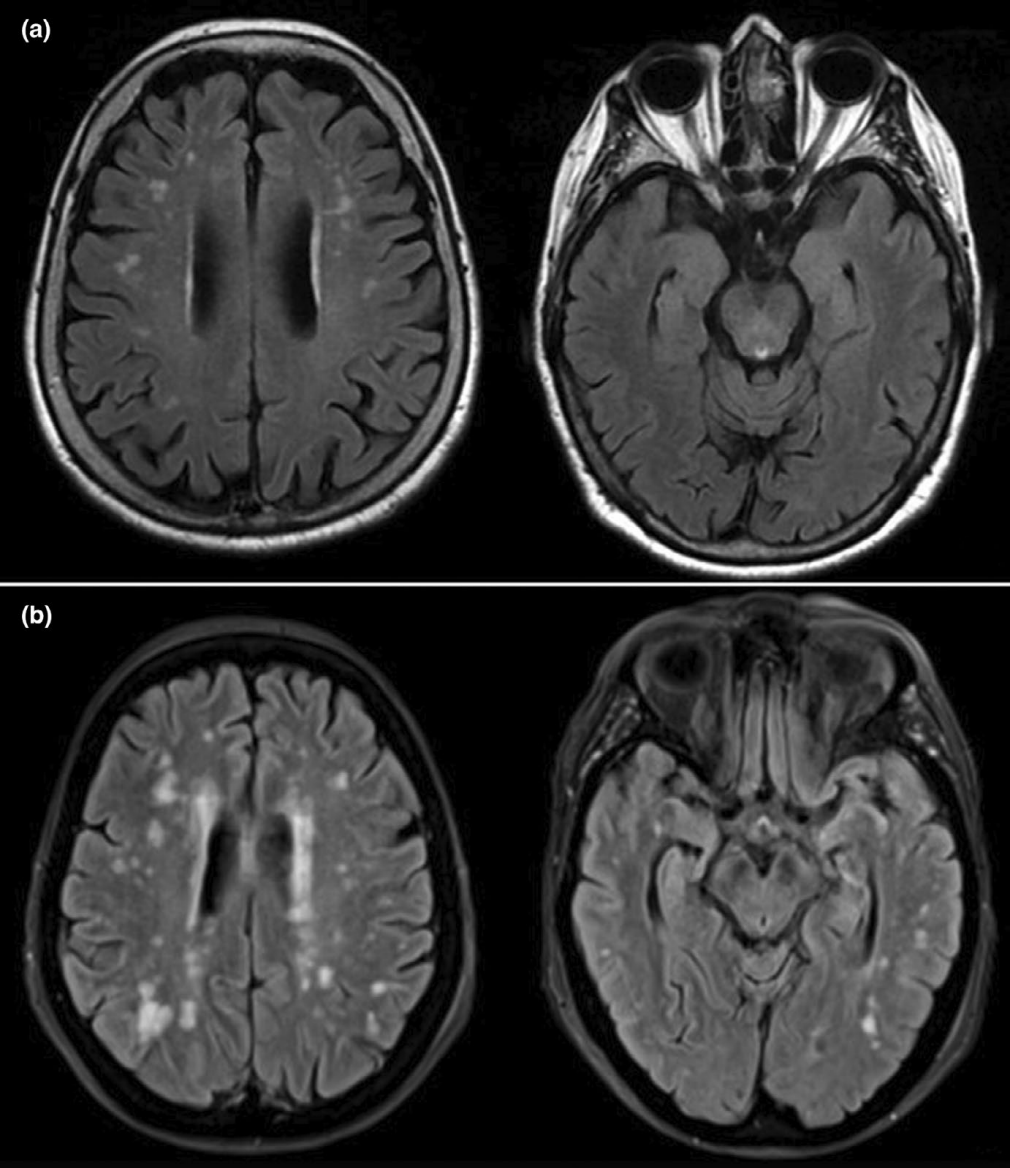
Axial T2 FLAIR MRI images demonstrating hyperintense lesions consistent with gliosis (a) in the right frontal and left temporal lobes; (b) in the frontal lobes.

Figure 2 displays the distribution and severity of white matter changes in the frontal and temporal lobes among the studied groups, visually comparing the ARWMC scores between G‐MCI and BMS‐MCI participants. Specifically, an elevated presence of white matter disease has been identified in these participants, quantified by the ARWMC score, with a focus on the frontal and temporal lobes in both groups.

### 3.6 Multilinear regression analyses

Table 7 summarizes multilinear regression analyses for BMS‐MCI, detailing how various predictors influence cognitive test scores. NRS negatively impacts RAVLT immediate recall (*p* = 0.015*), explaining 14.5% of variance suggests that pain severity directly detracts from verbal memory capabilities.

**TABLE 7:** Multilinear regression analysis predicting impaired cognitive test in the BMS‐MCI and G‐MCI groups.

BMS‐MCI				
Cognitive test	Predictors	β (SE)	*p*‐value	*R* ^2^ (*p*‐value)
MMSE	Essential Hypertension	−1.62 (0.83)	0.060	9.3 (0.060)
RAVLT immediate	NRS	−2.13 (0.84)	0.015*	**14.5 (0.015** * **)**
CGD	ESS	0.04 (0.05)	0.512	**13.2 (0.028** * **)**
	LT (Left Temporal)	−1.13 (0.46)	0.018*	
CB‐TT	RF (Right Frontal)	−0.23 (0.13)	0.101	6.9 (0.101)
TMT‐A	Years of education	−4.21 (1.38)	0.004**	**19.6 (0.004** ** **)**
TMT‐B	LF (Left Frontal)	41.7 (17.8)	0.024*	**12.6 (0.024** * **)**
TMTs‐Delta	NRS	12.95 (4.77)	0.010*	**35.3 (<0.001** ** **)**
	TC	1.00 (0.24)	<0.001**	
FAB	Years of education	0.12 (0.05)	0.030*	**16.3 (0.014** * **)**
	LDL	0.02 (0.01)	0.100	

*Note*: SE is the Standard Error of beta estimates. *p*‐values were obtained by hypothesis test on regression coefficients.

Abbreviations: BMS‐MCI, Burning mouth syndrome‐Mild cognitive impairment; CB‐TT, Corsi Block‐Tapping Task; CGD, Copying Geometric Drawings; ESS, Epworth Sleepiness Scale; FAB, Frontal Assessment Battery; HAM‐A, Hamilton Rating Scale for Anxiety; LDL, Low‐Density Lipoprotein; LF, Left Frontal; LPO, Left Parieto‐Occipital; LT, Left Temporal; MMSE, Mini‐Mental State Examination; PSQI, Pittsburgh Sleep Quality Index; RF, Right Frontal; RPO, Right parieto‐occipital; SF‐36, 36 items Short Form Survey; TC, Total cholesterol; TMTs, Trial Making Tests.

* Significant 0.01 < *p*‐value ≤0.05.

** Significant *p*‐value ≤0.01.

High ARWMC score in the LT area predict lower CGD scores (*p* = 0.018*), with a 13.2% variance explanation highlighting how localized WMCs can impair visuospatial abilities.

TMT‐A times decrease for each additional year of education (β = −4.21, *p* = 0.004**), accounting for 19.6% of variance emphasizing education's role in bolstering cognitive processing speed.

TMT‐B times increase by 41.7 s with a higher ARWMC score in LF (β = 41.7, *p* = 0.024*), explaining 12.6% of variance indicating that frontal white matter degeneration is particularly detrimental to tasks requiring cognitive flexibility.

NRS and TC levels significantly predict TMT‐Delta, increasing times by 12.95 (*p* = 0.010*) and 1.00 s per unit increase in TC (*p* < 0.001**), respectively, explaining 35.3% of variance suggesting that both metabolic health and pain management are crucial in maintaining cognitive functions in BMS‐MCI patients.

These results show that pain severity (NRS) emerges as a significant predictor of verbal memory performance, negatively impacting RAVLT immediate recall and highlighting the direct detrimental effect of chronic pain on cognitive functions. Additionally, WMCs in LT may affect visuospatial abilities, while in the frontal area significantly impairs cognitive flexibility. This suggests that specific regions of white matter degeneration are particularly impactful on certain cognitive domains.

Finally, education plays a protective role and enhances cognitive processing speed.

In G‐MCI patients (Table 8), the HAM‐A scores, along with MH and SF subscores of SF‐36, explain 38.2% of variance in MMSE scores (β = −0.27, *p* < 0.001**) suggesting anxiety's pervasive role in diminishing global cognitive function, while better mental health and social functioning enhance it.

**TABLE 8:** Multilinear regression analysis predicting impaired cognitive test in the G‐MCI group.

G‐MCI				
	Predictors	β (SE)	*p*‐value	*R* ^2^ (*p*‐value)
MMSE	HAM‐A	−0.27 (0.07)	<0.001**	**38.2 (<0.001** ** **)**
	SF‐36 MH	−0.01 (0.02)	0.622	
	SF‐36 SF	0.01 (0.02)	0.617	
CGD	Years of education	0.12 (0.07)	0.113	**25.7 (0.010** * **)**
	SF‐36 PF	0.02 (0.01)	0.182	
	SF‐36 BP	0.02 (0.01)	0.259	
	RPO (Right parieto‐occipital)	−0.04 (0.54)	0.947	
CB‐TT	PSQI	−0.07 (0.03)	0.070	**16 (0.033** * **)**
	SF‐36 RE	0.01 (0.01)	0.266	
	SF‐36 SF	0 (0.01)	0.569	
DCT	TC	0.1 (0.04)	0.015*	**14.6 (0.015** * **)**
TMT‐A	TC	−0.55 (0.19)	0.006**	**18.4 (0.006** ** **)**
TMT‐B	HAM‐A	3.58 (1.59)	0.030*	**21.7 (0.008** ** **)**
	TC	−0.59 (0.28)	0.039*	
	Atrial fibrillation	−86.6 (46.6)	0.071	
TMTs‐Delta	ESS	−4.99 (1.64)	0.005**	**31.9 (0.001** ** **)**
	HAM‐A	4.24 (1.66)	0.016*	
	SF‐36 MH	−0.33 (0.52)	0.534	
FAB	LPO (Left parieto‐occipital)	−1.98 (0.99)	0.052	**11.3 (0.042** * **)**
	Atrial fibrillation	3.93 (2.63)	0.144	

*Note*: SE is the standard error of beta estimates. *p*‐values were obtained by hypothesis test on regression coefficients.

Abbreviations: CB‐TT, Corsi Block‐Tapping Task; CGD, Copying Geometric Drawings; ESS, Epworth Sleepiness Scale; FAB, Frontal Assessment Battery; G‐MCI, Geriatric‐Mild cognitive impairment; HAM‐A, Hamilton Rating Scale for Anxiety; LDL, Low‐Density Lipoprotein; LF, Left Frontal; LPO, Left Parieto‐Occipital; LT, Left Temporal; MMSE, Mini‐Mental State Examination; PSQI, Pittsburgh Sleep Quality Index; RF, Right Frontal; RPO, Right Parieto‐Occipital; SF‐36, 36 items Short Form Survey; TC, Total cholesterol; TMTs, Trial Making Tests.

* Significant 0.01 < *p*‐value ≤0.05.

** Significant *p*‐value ≤0.01.

Education and ARWMC in the RPO area are positively associated with CGD performance, explaining 25.7% of variance (*p* = 0.010*) highlighting the protective effects of education against spatial and motor skill decline.

PSQI, RE, and SF of SF‐36 correlate with CB‐TT, explaining 16% of variance (*p* = 0.033*).

Therefore, sleep quality and emotional/social functioning, without individually significant predictions highlight how well‐being dimensions influence working memory and executive function.

TC levels inversely correlate with TMT‐A (β = −0.55, *p* < 0.006**), explaining 18.4% of TMT‐A variance and positively correlate with TMT‐B (β = 3.58, *p* < 0.030**), explaining 21.7% of TMT‐B variance suggesting cholesterol's complex impact on processing speed and task‐switching abilities, possibly indicating differential effects based on cognitive demand and task nature.

## 4 DISCUSSION

BMS has not been directly linked to cognitive decline conditions such as dementia and Parkinson's disease. The significant disparities in cognitive functions between BMS‐MCI and G‐MCI patients, underline the heterogeneity of MCI. Therefore, the marked decline in specific cognitive domains in BMS‐MCI patients may be related to a different pathological mechanism compared to G‐MCI patients. A retrospective analysis conducted by Kim et al. revealed that the incidence rates of dementia and Parkinson's disease remained comparable between BMS patients and controls (Kim et al., 2020). Contrasting these findings, recent research by Canfora et al. presents a compelling case for a potential connection between BMS and cognitive deterioration (Canfora et al., 2021). This case–control study revealed cognitive impairments in BMS patients, particularly in attention, working memory, and executive functions, compared to age and gender‐matched controls. These findings suggest a potential link between BMS and cognitive decline, possibly increasing neurodegenerative disorder risk. Identified cognitive impairment patients were categorized as having MCI. Ongoing research on MCI subtypes aims to delineate unique diagnostic and prognostic features. This study aims to compare cognitive and psychological profiles of BMS patients with MCI to age and gender‐matched MCI patients without BMS, refining classification, and prognosis.

In this study, global cognitive function, as measured by the MMSE, exhibited a pronounced decline in BMS‐MCI patients compared to G‐MCI patients. This indicates a substantial global cognitive deficit within the BMS‐MCI group, suggesting a more severe impairment in this cohort. Similarly, the higher scores of TMT‐A in BMS‐MCI compared with G‐MCI underscores a marked impairment in visual attention and processing speed among individuals suffering from BMS alongside cognitive decline. However, no significant differences in TMT‐B and TMT‐delta scores across both BMS‐MCI and G‐MCI groups may suggest comparable levels of executive dysfunction, reflecting challenges in cognitive flexibility, task‐switching, and handling complex cognitive loads. This finding suggests that, despite the additional burden of BMS in the BMS‐MCI group, the fundamental nature of executive function impairment remains consistent across different manifestations of MCI. Therefore, the executive dysfunction observed in MCI appears to be a robust feature that transcends specific patient subgroups, including those with concurrent chronic conditions like BMS.

Regarding visuo‐spatial working memory, as suggested by the score of CB‐TT, a comparable level of function was found between BMS‐MCI and G‐MCI patients. This indicates that, despite differences in other cognitive areas, the ability to temporarily maintain and manipulate information in working memory does not significantly differ between the two groups.

Conversely, BMS‐MCI patients exhibited significantly higher scores in both immediate and delayed recall compared to G‐MCI patients highlighting substantial impairments in verbal memory among the G‐MCI patients. This suggests better verbal memory performance in the BMS‐MCI groups, while G‐MCI showed more severe issues in the ability to encode, retain, and retrieve verbal information.

No statistical differences were found in scores for the FAB, CGD, and DCT between the two groups, indicating that, despite deficits in processing speed, attentional control, and cognitive flexibility, visuo‐spatial abilities, and focused attention are preserved.

From the analysis of these data, it is clear that a greater impairment in global cognitive function, attention, and processing speed in BMS‐MCI patients compared to those with G‐MCI, while working memory, executive functions, and visuo‐motor coordination do not show significant differences between the groups. Instead, global cognitive functions are relatively preserved in G‐MCI compared to BMS‐MCI with notable deficits in verbal memory.

The lack of significant differences in sociodemographic characteristics and risk factors like smoking, hypertension, and dyslipidemia between BMS‐MCI and G‐MCI patients suggests that these factors alone do not explain the variations in cognitive decline observed between the two groups. Conversely, BMS‐MCI exhibited more pronounced psychological distress within the BMS‐MCI group as suggested by higher scores on the HAM‐D and HAM‐A. Several studies have reported that anxiety, depression, and cognitive decline are strictly interconnected (Goulabchand et al., 2022; Ma, 2020). Indeed, mood disorders have been associated with an increased risk for cognitive decline and cognitive impairments are common in both major depression and anxiety disorders, affecting executive function, memory, and information processing (Marvel & Paradiso, 2004). The nature of these deficits, however, may vary depending on disorder characteristics and subtypes. Executive dysfunction is evident in major depression, while specific anxiety disorder subtypes, such as obsessive‐compulsive disorder, are associated with deficits in executive functioning and visual memory (Castaneda et al., 2008). In addition, the presence of depressive symptoms has been identified as an independently predictive factor of conversion from MCI to AD (Gallagher et al., 2011; Gulpers et al., 2016; Rozzini et al., 2008).

However, from the analysis of correlation and multilinear regression, anxiety and depression were not predictors of cognitive decline in BMS‐MCI, conversely, anxiety emerged as a significant predictor of cognitive performance only in G‐MCI. Indeed, anxiety was a predictor of MMSE, TMT‐B, and the TMT‐Delta suggesting that heightened anxiety levels may detrimentally influence both general cognitive status and more sophisticated cognitive functions such as executive control, cognitive flexibility, and task‐switching capabilities in this group. From the analysis of current literature, specifically, anxiety negatively affects the prefrontal cortex, leading to diminished executive function and mental flexibility, attributes critically assessed by TMT‐B. For instance, Uemura and colleagues and Klojčnik et al. provide empirical support for this adverse impact of anxious symptoms on executive functioning (Klojčnik et al., 2017; Uemura et al., 2014). On the contrary, MacPherson and his team discovered that age‐related declines in processing speed significantly affect executive function, as determined by TMT‐B performance, regardless of the presence of anxiety (Macpherson et al., 2017).

The lack of correlation between mood disorders and cognitive tasks in BMS‐MCI may further confirm that cognitive decline in BMS patients may be related to different factors as demonstrated by regression analysis.

Specifically, pain was a significant predictor affecting verbal memory (RAVLT immediate) and tasks requiring cognitive flexibility or the ability to rapidly switch from one type of activity to another (TMT‐delta). These results confirm previous experimental and clinical studies. Cohen et al have demonstrated that rats with induced pain showed impaired performance in tasks requiring cognitive flexibility, such as selecting high‐reward options, suggesting that pain can lead to significant alterations in learning and decision‐making strategies (Cohen et al., 2018). Moreover, structural white matter abnormalities in pain‐modulating regions may disrupt cognition required for effective executive control and attentional performance (Gomez Beldarrain et al., 2005). These changes can negatively impact the brain's capacity to perform complex mental activities, thereby reducing cognitive reserve. Moreover, chronic pain requires constant cognitive effort for its management, which can reduce the cognitive resources required for effective executive control and attentional performance (Moore et al., 2012).

Notably, statistically significant differences were observed in the right and left temporal regions in individuals with G‐MCI compared to those with BMS‐MCI, suggesting that WMCs may play a role in specific cognitive functions. This study suggests that differences in white matter changes, particularly in the temporal regions, may influence distinct cognitive abilities in individuals with different types of MCI. Previous research has demonstrated that alterations in white matter integrity, particularly in tracts connecting the temporal and frontal lobes, can impact specific cognitive abilities such as cognitive flexibility, processing speed, and executive functions (Kullmann et al., 2015; Walterfang et al., 2008). Moreover, WMCs in the temporal area may serve as a precursor to temporal lobe atrophy, a risk factor for AD, and are generally associated with impairments in executive functions and mental flexibility. Various studies underscore the significance of temporal white matter changes in the early stages of cognitive decline and their link with the progression to AD (Cordonnier & van der Flier, 2011; Prins & Scheltens, 2015). Furthermore, these changes in the temporal lobe, leading to structural alterations in brain regions involved in memory processing (Barone et al., 2018; Lancaster et al., 2016), may indirectly influence verbal memory deficits, contributing to temporal lobe atrophy. Such findings could elucidate the observed impairment in verbal memory among G‐MCI patients, as indicated by their lower performance on both immediate and delayed RAVLT scores compared to those with BMS‐MCI (Swardfager et al., 2018).

From the analysis of regression, higher TC levels are linked to performance in tasks such as the TMT‐Delta in BMS‐MCI individuals and with DCT, TMT‐A, and TMT‐B in G‐MCI individuals. These findings suggest a potential link between elevated cholesterol levels and cognitive task performance, underscoring the importance of considering lipid profiles in cognitive health assessments. The relationship between TC levels and cognitive function, specifically executive function, has been explored in various studies with mixed findings. For instance, Gendle et al. found that elevated TC levels were negatively associated with executive control and sustained attention, suggesting that higher cholesterol levels could impair certain cognitive functions (Gendle et al., 2008). Conversely, Elias et al. reported that lower naturally occurring TC levels were linked to poorer performance on cognitive measures requiring high executive functioning, indicating that not just high, but also suboptimal low cholesterol levels could be detrimental to cognitive health (Elias et al., 2005).

Furthermore, the review of Anstey et al. introduces an age‐dependent perspective, highlighting that high midlife TC levels are associated with an increased risk of AD and dementia, yet no such association is found with late‐life TC levels (Anstey et al., 2017). This suggests that the timing and duration of cholesterol exposure are crucial factors in understanding its impact on cognitive decline and dementia risk.

SF‐36 analysis reveals a link between quality of life and cognitive decline in G‐MCI patients. Even when PF and MH domains are not individually significant, their combined influence correlates with cognitive test outcomes, indicating that improved physical and mental health may benefit cognitive function in this group.

Finally, in line with previous studies years of education significantly impacted on cognitive test outcomes suggesting that higher educational attainment may be associated with better cognitive performance (Dufouil et al., 2003; Lövdén et al., 2020). In BMS‐MCI, each extra year of education correlates with a notable decrease in TMT‐A completion time, indicating improved processing speed and executive function. These results underscore education's protective effect on cognitive functions, affirming its role in building cognitive reserve.

These results distinguish between two MCI groups: G‐MCI and BMS‐MCI, based on their cognitive profiles. G‐MCI exhibits an “amnestic profile” with significant memory impairment, unlike BMS‐MCI. Amnestic MCI (aMCI) primarily affects memory, while non‐amnestic MCI impacts other cognitive functions like attention, executive function, and language skills. Individuals with aMCI face higher AD risk compared to those without memory issues or non‐amnestic MCI, aligning with literature indicating memory problems as early signs of AD.

Analyzing cognitive assessments reveals that BMS‐MCI predominantly maintains memory functions but shows significant impairments in other cognitive areas, aligning more with non‐amnestic MCI. Core challenges involve executive functions, attention, and general cognitive processing. This classification as multiple domain non‐amnestic MCI is supported by impairments in global cognitive functions, attention, and executive function. Despite compromised cognitive capacity, BMS‐MCI patients may have a lower risk of developing AD. The findings from our study suggest that it would be advisable for clinicians to screen for cognitive and neuropsychological functions in older patients with BMS, in order to early detect early MCI and plan a treatment plan. Multidimensional interventions involving diet, lifestyle modification, and cognitive stimulation have proven particularly effective at improving executive functions in populations at risk (Ngandu et al., 2015).

### 4.1 Limitations

The study's cross‐sectional design limits definitive conclusions on BMS and cognitive impairments. Results are exploratory; caution in interpretation is advised. Longitudinal studies are recommended for understanding cognitive decline trajectory in BMS patients. With 40 participants per group, initial observations were possible, but larger samples are crucial for confirmation and generalizability. While age, gender, and education were matched, uncontrolled variables like lifestyle, nutrition, and genetics could impact outcomes and warrant isolation in future studies. Moreover, other comorbid conditions of the elderly were not reported in this study, but accounting for them in future reports might give further insight on the role of comorbidities in the BMS cognitive profile.

## 5 CONCLUSIONS

This study has shed light on the intricate relationship between BMS and cognitive impairment, challenging previously existing paradigms and significantly advancing our comprehension of how chronic conditions impact cognitive health. By comparing cognitive decline in patients with BMS to those without, our analysis not only confirms the presence of specific cognitive deficits associated with BMS but also suggests a more complex interplay at work than previously recognized. These results could lead to the categorization of BMS‐MCI predominantly within the non‐amnestic subtype and G‐MCI within the amnestic subtype, the latter associated with a higher risk of AD.

Additionally, the white matter damage, particularly within the temporal and frontal regions, may have potentially contributed to this categorization.

These distinctions emphasize not only the complexity of cognitive impairment in MCI but also underscore the importance of nuanced diagnostic and therapeutic integrated approaches.

A holistic, multifaceted approach addressing education, mental health, metabolic and cardiovascular health, pain management, and sleep quality is essential for mitigating cognitive decline and improving patient outcomes.

Tailored cognitive rehabilitation programs, particularly those aimed at improving cognitive flexibility and problem‐solving skills in BMS sufferers, could significantly enhance patient outcomes, enriching their quality of life and potentially decelerating cognitive decline. Advocating for interventions that bolster cardiovascular and physical health through lifestyle modifications is essential for promoting healthy aging. Such measures are pivotal not just for reinforcing the brain's resilience and neuroplasticity but also for indirectly slowing the progression of WMCs.

Further research is needed to explore the underlying mechanisms linking cognitive decline, WMCs, and metabolic health and to investigate cognitive impairment in other chronic pain conditions. Longitudinal studies could provide more insights into the progression of cognitive impairments and the long‐term effects of educational and lifestyle interventions.

## AUTHOR CONTRIBUTIONS

**Grazia Daniela Femminella:** Conceptualization; investigation; methodology; validation; writing – review and editing; project administration; supervision. **Federica Canfora:** Conceptualization; investigation; writing – original draft; methodology; validation; visualization; writing – review and editing; software; data curation; resources. **Gennaro Musella:** Conceptualization; investigation; writing – original draft. **Gianluca Scotto Di Tella:** Writing – original draft; methodology; validation. **Lorenzo Ugga:** Writing – original draft; writing – review and editing; visualization. **Giuseppe Pecoraro:** Conceptualization; validation; methodology; data curation. **Stefania Leuci:** Conceptualization; methodology; data curation. **Noemi Coppola:** Conceptualization; methodology; data curation. **Natascia De Lucia:** Conceptualization; methodology; data curation. **Nelson Mauro Maldonato:** Validation; conceptualization; data curation; supervision. **Simone Liguori:** Data curation; methodology. **Massimo Aria:** Software; formal analysis; project administration; writing – review and editing; writing – original draft. **Luca D'Aniello:** Software; formal analysis; project administration; data curation; writing – review and editing; writing – original draft. **Giuseppe Rengo:** Resources; supervision; conceptualization; visualization; project administration. **Michele Davide Mignogna:** Resources; supervision; project administration; methodology; conceptualization. **Daniela Adamo:** Resources; supervision; conceptualization; investigation; writing – original draft; methodology; validation; visualization; writing – review and editing; software.

## CONFLICT OF INTEREST STATEMENT

No conflicts of interest to report.

## PATIENT CONSENT STATEMENT

Written informed consent was obtained from all subjects involved in the study and no financial compensation was given for participation.

## Supporting information


Tables S1‐S3

## Data Availability

The data can be obtained from the corresponding author upon request.
