# Supplementary material for: Cognitive profile in burning mouth syndrome versus mild cognitive impairment: A comparative study
Source: Oral Dis. 2024 Jul 30;31(2):611–32. doi: 10.1111/odi.15087 (PMC11976131; doi:10.1111/odi.15087)
Supplement: Supplementary file 1 — Tables S1‐S3 [file ODI-31-611-s001.docx]

**Table S1: Psychological, Sleep and Pain Assessment**

| **Test** | **Description** |
| --- | --- |
| Hamilton Depression scale (HAM-D)  (Hamilton, 1960) | 21 items where a scores exceeding 7 indicates impairment; scores between 7 and 17 suggest mild depression, 18 to 24 indicate moderate depression, and scores exceeding 24 indicate severe depression. |
| Hamilton Anxiety Scale (HAM-A)  (Hamilton, 1959) | Scores ranging from 0 to 4 for a total of 14 items. A cumulative score below 17 suggests mild severity, 18 to 24 indicates mild to moderate severity, and 25 to 30 suggests moderate to severe severity. |
| 36-Item Short Form Survey (SF-36)  (Lins and Carvalho, 2016) | It is made up of eight health domains, 4 for physical health and 4 for mental health, and is a collection of general, consistent, and simple-to-administer quality-of-life indicators. The four scales that comprise the physical components dimension are bodily pain (BP) with two items, role physical (RP) with four items, physical functioning (PF) with ten things, and general health (GH) with five items. Conversely, the mental components dimension consists of four questions for vitality (VT), two for social functioning (SF), three for role emotional (RE), and five for mental health (MH). The scores on the SF-36 scales range from 0 to 100. A mean score of 50 has been established as the normative value for all measures, with higher scores indicating better health condition. |
| Pittsburgh Sleep Quality Index (PSQI)  (Curcio *et al*, 2013) | 18 items that included both Likert-type and open-ended questions, was used to measure the quality of sleep. Each question has a score between 0 and 3, where higher scores correspond to more severe sleep disruptions. Individuals with poor and good sleep quality can be distinguished with high sensitivity and specificity using a global scale cut-off of 5. |
| Epworth Sleepiness Scale (ESS)  (Johns, 1991) | Self-administered questionnaire with 8 questions, was used to measure daytime sleepiness. Respondents are asked to rate, on a 4-point scale (0-3), their usual chances of dozing off or falling asleep while engaged in eight different activities. A score over 10 indicates notable daytime sleepiness. |
| Numeric Rating Scale (NRS)  (Boonstra *et al*, 2016) | A scale from 0 to 10, where 0 represents no intensity or magnitude, and 10 represents the highest possible intensity. |
| Short form of the McGill Pain Questionnaire (SF-MPQ)  (Melzack, 1987) | a range between 0-45 derived by the sum of all the 15 item scores, each rated from 0 (none) to 3 (severe). |

**Table S2: Age Related White Matter Changes (ARWMCs) characteristics**

| four-point scale | score 0 = no lesions, 1 = focal lesions; 2 = beginning confluence of lesions; and 3 = diffuse involvement) |
| --- | --- |
| results can be presented as the total score and global score | the total score was used, representing the sum of scores for each region in both hemispheres, which range from 0 to 30.* |

(Wahlund *et al*, 2001)

*Lesions in the basal ganglia were ranked in the same way and counted as white matter lesions even if found in the gray matter nuclei, which includes a small amount of white matter.

**Table S3: Neurocognitive Assessment Tests**

| **Domain** | **Test** | **Description** |
| --- | --- | --- |
| **Global cognitive function** | Mini Mental State Examination  (MMSE)  (Roselli *et al*, 2009) | MMSE evaluates cognitive functions; it includes tests of orientation, attention, short-term memory, language and visual-spatial skills. |
| **Attention** | Trial Making A  (TMT-A)  (Tombaugh, 2004) | Measure of attention, speed and mental flexibility through connecting numbers from 1 to 25 in ascending order. |
|  | Digit Cancellation Test  (DCT)  (Hatta *et al*, 2012) | Evaluation of focused, sustained and selective attention. It includes three different matrices with one, two and three target digits. Subject is asked to cross out the corrected targets in 45 seconds per matrix. |
| **Working Memory** | Corsi block-tapping task  (CB-TT)  (Berch *et al*, 1998) | Measure of [visuo-spatial](https://en.wikipedia.org/wiki/Visual-spatial_ability) [short term working memory](https://en.wikipedia.org/wiki/Short_term_memory). Subject is asked to point at the blocks in the order they are tapped. |
| **Verbal memory** | Rey Auditory Verbal Learning Test (RAVLT)  (King *et al*, 1998) | Measure of verbal learning and memory. Subject is asked to repeat a list of 15 words for 5 consecutive trials; there is the immediate recall and a 15-min delayed recall. |
| **Constructional apraxia** | Copying Geometric Drawings  (CGD)  (Bat *et al*, 2022) | Evaluation of constructional apraxia. The task is composed of 7 tables to copy in the same way. |
| **Executive Function** | Trail Making B  (TMT-B)  (Tombaugh, 2004) | Measure of mental flexibility. Subject is asked to connect alternating numbers (1–13) and letters (A-L). |
|  | Frontal Assessment Battery  (FAB)  (Appollonio *et al*, 2005) | Measure of executive function to assess the functions in the frontal lobe. It includes six sub-items. |

**References**

Appollonio I, Leone M, Isella V, *et al* (2005). The Frontal Assessment Battery (FAB): normative values in an Italian population sample. *Neurol Sci* **26**: 108–116.

Bat BKK, Chan JYC, Chan TK, *et al* (2022). Comparing drawing under instructions with image copying for mild cognitive impairment (MCI) or dementia screening: a meta-analysis of 92 diagnostic studies. *Aging Ment Health* **26**: 1019–1026.

Berch DB, Krikorian R, Huha EM (1998). The Corsi Block-Tapping Task: Methodological and Theoretical Considerations. *Brain and Cognition* **38**: 317–338.

Boonstra AM, Stewart RE, Köke AJA, *et al* (2016). Cut-Off Points for Mild, Moderate, and Severe Pain on the Numeric Rating Scale for Pain in Patients with Chronic Musculoskeletal Pain: Variability and Influence of Sex and Catastrophizing. *Front Psychol* **7**: 1466.

Curcio G, Tempesta D, Scarlata S, *et al* (2013). Validity of the Italian version of the Pittsburgh Sleep Quality Index (PSQI). *Neurol Sci* **34**: 511–519.

Hamilton M (1959). The assessment of anxiety states by rating. *Br J Med Psychol* **32**: 50–55.

Hamilton M (1960). A rating scale for depression. *J Neurol Neurosurg Psychiatry* **23**: 56–62.

Hatta T, Yoshizaki K, Ito Y, Mase M, Kabasawa H (2012). RELIABILITY AND VALIDITY OF THE DIGIT CANCELLATION TEST, A BRIEF SCREEN OF ATTENTION. *An International Journal of Psychological Sciences* **55**: 246–256.

Johns MW (1991). A new method for measuring daytime sleepiness: the Epworth sleepiness scale. *Sleep* **14**: 540–545.

King JH, Gfeller JD, Davis HP (1998). Detecting Simulated Memory Impairment with the Rey Auditory Verbal Learning Test: Implications of Base Rates and Study Generalizability. *Journal of Clinical and Experimental Neuropsychology* **20**: 603–612.

Lins L, Carvalho FM (2016). SF-36 total score as a single measure of health-related quality of life: Scoping review. *SAGE Open Med* **4**: 2050312116671725.

Melzack R (1987). The short-form McGill Pain Questionnaire. *Pain* **30**: 191–197.

Roselli F, Tartaglione B, Federico F, Lepore V, Defazio G, Livrea P (2009). Rate of MMSE score change in Alzheimer’s disease: Influence of education and vascular risk factors. *Clinical Neurology and Neurosurgery* **111**: 327–330.

Tombaugh TN (2004). Trail Making Test A and B: normative data stratified by age and education. *Arch Clin Neuropsychol* **19**: 203–214.

Wahlund LO, Barkhof F, Fazekas F, *et al* (2001). A new rating scale for age-related white matter changes applicable to MRI and CT. *Stroke* **32**: 1318–1322.
